# Task-unrelated thought increases after consumption of COVID-19 and general news

**DOI:** 10.1186/s41235-022-00420-7

**Published:** 2022-07-25

**Authors:** Chelsie M. Hart, Caitlin Mills, Raela F. Thiemann, Jessica R. Andrews-Hanna, Lianne Tomfohr-Madsen, Julia W. Y. Kam

**Affiliations:** 1 Department of Psychology, University of Calgary, 2500 University Drive, Calgary, AB T2N 1N4 Canada; 2 Department of Psychology, University of New Hampshire, 15 Academic Way, Durham, NH 03824 USA; 3 Department of Psychology, Cognitive Science, University of Arizona, 1503 E University Blvd, Tucson, AZ 85721 USA; 4 Hotchkiss Brain Institute, University of Calgary, 3330 Hospital Drive NW, Calgary, AB T2N 4N1 Canada

**Keywords:** Task-unrelated thought, News, Coronavirus, Ecological momentary assessment

## Abstract

Task-unrelated thoughts (TUTs) are frequent distractions from our everyday tasks, which can reduce productivity and safety during task performance. This necessitates the examination of factors that modulate TUT occurrence in daily life. One factor that has previously been implicated as a source of TUT is personally salient concerns. External factors such as news media serve as cues for these concerns, potentially increasing TUT occurrence. However, this has not been thoroughly examined in everyday life settings. We thus utilized Ecological Momentary Assessment to survey participants throughout the day for ten days, on their TUTs and news consumption in two separate studies. Study 1 focused on the impact of news related to the Coronavirus Disease (COVID-19) pandemic, as a common and global concern. We found that TUT occurrence was significantly predicted by COVID-19 news consumption, such that TUT occurrence increased with COVID-19 news consumption. To extend these findings, we implemented Study 2 using similar methods, but focusing on the consumption of news media in general. TUT occurrence was predicted by general news consumption, with an increase in reports of TUT following consumption of news media in general. We thus extended the association found between TUT and COVID-19-related news in Study 1, to any news topic in Study 2. We speculate that the increase in TUTs was due to heightened salience of current concerns, cued by the news. These findings highlight the importance of considering when we choose to consume news media and the value of examining contextual factors when studying TUTs in daily life.

**Supplementary Information:**

The online version contains supplementary material available at 10.1186/s41235-022-00420-7.

## Significance Statement

Task-unrelated thoughts (TUTs) are common in everyday life. They are exemplified by experiences like planning your day while getting ready in the morning or thinking about dinner when driving home from work. Not only are TUTs common, but they can also be disruptive during daily tasks requiring our focus. To prevent the disruptive nature of TUTs requires a better understanding of what factors can impact their occurrence. Accordingly, we ran two studies to examine the unique impact of news consumption on TUT occurrence. In Study 1, we specifically examined consumption of news related to the Coronavirus Disease (COVID-19) pandemic, as a current issue that relates to personally salient concerns. Results showed that consuming news related to COVID-19 was associated with greater TUT occurrence. We expanded on these results in Study 2, where we found that general news media consumption was associated with greater TUT occurrence as well. These studies suggest the importance of considering daily news consumption patterns when trying to avoid unwanted TUTs. Increasing awareness of factors that influence our attentional focus is an important step toward improving our ability to focus on the task-at-hand in order to complete it safely and efficiently.

## Introduction

The human mind spends a great deal of time on thoughts unrelated to current tasks, known as task-unrelated thoughts (TUTs; Smallwood & Schooler, 2015). Past research has highlighted the benefits of TUTs via their role in important functions such as future planning (Baird et al., 2011) and creativity (Baird et al., 2012). However, TUTs can also disrupt task performance in daily life (e.g., Kam & Handy, 2014; McVay et al., 2009). This has been shown on the road while driving (He et al., 2011), as well as in educational or occupational settings (e.g., Risko et al., 2012; Seli et al., 2016). These adverse outcomes necessitate the examination of factors that influence TUT occurrence in ecological settings with the aim to reduce its negative impact in our lives. Accordingly, our goal was to examine factors that may change the frequency of TUTs in daily life based on prominent theoretical assumptions about the nature of TUT—specifically, its relationship to current concerns (Klinger & Cox, 2011).

Common factors that modulate our TUTs are our goals and concerns: when we have unresolved goals, TUT occurrence increases. Indeed, ample research has shown that TUTs are often focused on our unresolved goals, referred to as current concerns (e.g., Geerligs, 1995; Smallwood et al., 2009). According to Klinger and Cox’s (2011) Motivation and Goal Theory of Current Concerns, humans evolved to form and pursue goals. If our ongoing tasks prevent us from actively addressing our current concerns, our mind engages in TUTs to mentally address them until we can act on them (Klinger, 2013). Empirical evidence from laboratory studies suggests that TUTs are related to our current concerns, particularly when these concerns become salient following exposure to cues that remind us of them (e.g., Kopp et al., 2015; McVay & Kane, 2013). However, these studies offer tightly controlled manipulations of current concerns through cues (e.g., having participants create to-do lists prior to a laboratory task, embedding concern-related stimuli in tasks), which do not necessarily capture how such concerns may manifest in everyday life.

In today’s world, a common external source of information that increases the salience of our concerns is the news. Much of the news through 2020 and 2022 has focused on the coronavirus (COVID-19) pandemic. Not only have individuals been worried about the direct consequences of COVID-19 on physical and mental health, but they have also been concerned about their education, employment, travel, and many other aspects of life (Gromada et al., 2020; Jun et al., 2021). Thus, news related to COVID-19 has been directly relevant to many current concerns. In this manner, the high salience of the pandemic and constant reminders about its presence via different news outlets may impact our propensity for TUTs in daily life. Indeed, a fabricated news broadcast about America going to war with China has previously been shown to increase rates of TUT occurrence in an experimental setting (Antrobus et al., 1966). The content of these TUTs was specifically related to the consequences of this potential war and war in general. While this demonstrates how a single fake news broadcast can elicit personal concerns that became the focus of participants’ thoughts during experimental tasks, less known is the impact of news exposure on the occurrence of TUTs in ecological settings.

One recent study from McKeown and colleagues (2021) sampled participants’ ongoing experiences multiple times a day, using a method often referred to as Ecological Momentary Assessments (EMA; Shiffman et al., 2008). For one week before and one week during a lockdown to reduce the transmission of COVID-19, participants filled out brief surveys on their thoughts five times a day. They found that, while the lockdown did not affect how often people were focused on their tasks, thought patterns changed while people were consuming media (comprising of social media, television/film, music, radio/podcasts, and news media). Specifically, thoughts related to future plans, goals, and current tasks were reduced while consuming media, and imagery-focused thoughts were increased. While this effect was not specific to news media, these findings provide preliminary evidence that the effect of news has the potential to impact our thought patterns in everyday life as measured by EMA.

Other studies have also demonstrated the utility of EMA in assessing TUTs (e.g., Kane et al., 2017) and current concerns (e.g., De Leersnyder et al., 2018). By randomly sampling individuals’ in-the-moment experiences, EMA captures contextual changes in ecologically valid settings and effectively avoids issues of recall bias and accuracy that are often seen in retrospective measures of TUT (Shiffman et al., 2008). Thus, we used EMA as a fine-grained measure of when TUTs and news consumption occur throughout the course of the day. Specifically, we examined the dynamic changes in TUT occurrence related to COVID-19 news consumption in Study 1 and general news consumption in Study 2.

Based on past findings and the Motivation and Goal Theory of Current Concerns (Klinger & Cox, 2011), we hypothesized that consumption of COVID-19 news would be associated with an increase in TUT occurrence in Study 1, and that similar patterns would be shown for news in general in Study 2. Given the observational nature of the EMA methodology, we cannot infer causal direction; however, demonstrating a relationship between news media and TUTs in daily life will highlight the importance of considering context in understanding these thoughts. As greater task motivation has been shown to reduce TUT occurrence (e.g., Giambra & Grodskey, 1989; Robison & Unsworth, 2018) and as motivation is part of our pursuit of current concern-related goals (Klinger & Cox, 2011), we also examined whether motivation moderated the effect of news consumption on TUT occurrence, to further contextualize this relationship.

## Study 1 methods

The data used for Study 1 were originally gathered as part of a study examining the potential for mindfulness training as an intervention to reduce the negative impacts of the COVID-19 pandemic on mental health (Kam et al., 2021). The study was approved by the Conjoint Faculties Research Ethics Board at the University of Calgary.

### Participants

Sixty-four healthy participants were recruited on a rolling basis via emails sent to graduate programs at, and communities connected to, several universities in North America. Data collection occurred from May 6th to May 31st, 2020. During this time, we are not aware of any major changes in policy or unexpected events that would impact all participants simultaneously. Participant recruitment ended as new COVID-19 cases began to plateau in Alberta, Canada–the primary site of recruitment. Decreases or plateaus in new COVID-19 cases were also shown in other areas of recruitment at this time (e.g., British Columbia, Canada; New Hampshire, USA). To be included in the study, participants were required to be between 17 and 65 years of age, fluent in English, and to not have practiced mindfulness more than once a week in the past three months. Two participants did not complete the study, reducing the sample to 62 participants. Further exclusions were applied based on EMA data criteria (described in more detail below), resulting in a final sample of 58 participants; this is comparable to similar studies on mindfulness training (Economides et al., 2018). Participants were 29.97 years old on average (SD = 9.05, range: 19 to 63 years); most identified as female (48, 82.8%), and as white^1^ (36, 62.1%) or Asian (15, 25.9%).

In the original study, participants were randomly assigned to mindfulness training or waitlist control groups (Kam et al., 2021). These groups did not differ on TUT occurrence assessed via EMA, as shown in Additional file 1: Supplementary Table 1, and including mindfulness training group as a covariate in our regression models examining the impact of COVID-19 news consumption on TUT did not alter our conclusions for the current study. Further, the two groups did not meaningfully differ on questionnaire-based measures of TUT taken before and after the training period, as shown in Additional file 1: Supplementary Tables 2 and 3. Given these three findings converge on the notion that mindfulness training did not impact TUT occurrence or the outcomes involving COVID-19 news consumption, we collapsed across the two groups in our subsequent analyses.

### Procedure

In this 12-day study, day 1 began with participants completing informed consent, followed by a demographics survey which included questions about previous experiences with mindfulness training and frequency of COVID-19-related news consumption. They then filled out a set of questionnaires as baseline measures for the purpose of evaluating the benefits of mindfulness in the original study (Kam et al., 2021). The full list of questionnaires implemented are reported in the Supplementary Materials and will not be described further here.

Like many EMA studies of TUT (e.g., Kane et al., 2017; McVay et al., 2009; Mills et al., 2018) participants in this study also completed an instructions and exercise survey on day 1. The instructions described information regarding the EMA survey in detail, including definitions for terminology and examples to illustrate the meaning of different responses. They were then given an exercise where they were asked to complete an EMA survey based on a given scenario to ensure they understood the instructions before proceeding in the study. The full training instructions as well as the scenario from the exercise are available in Additional file 1: Supplementary Table 4. The mindfulness training and main EMA portion of the study spanned days 2 to 11. On day 12, participants completed the same set of questionnaires from day 1, as part of the evaluation of the impact of mindfulness (Kam et al., 2021) which is not relevant to the current study and therefore will not be reported further.

### Measures–Ecological Momentary Assessment

All participants received four EMA surveys daily for ten days (i.e., day 2 to day 11), for a total of 40 surveys. These were sent randomly via email during a 10-hr time window of participants’ choice to best capture individual circadian rhythms, which were likely adjusted during the pandemic due to lifestyle changes such as working from home. To ensure timeliness and validity of the EMA responses in capturing the in-the-moment experience, participants were asked to allow email notifications on their phones and to only respond to the survey prompts if it was possible to do so safely within 15 min of receiving the email. Additionally, participants were instructed to report on their thoughts from the moment just before receiving the prompt, rather than their thoughts in general.

Each EMA survey consisted of the same set of questions shown in the training session on day 1. The full list of questions is outlined in Appendix Table 3. Question order was not randomized or counterbalanced to minimize response time and participant confusion, as well as to reduce the disruption EMA surveys may cause when interrupting tasks. Study 1 focused specifically on participants’ TUT ratings (“How on-task [task-related] or off-task [task-unrelated] was your thought?” responded on a scale from 1 = “completely on-task” to 4 = “extremely off-task”), task motivation (“How motivated were you to do the [current] task?” responded on a scale from 1 = “not motivated at all” to 5 = “extremely motivated), and whether they had consumed COVID-19-related news in the two hours before doing the survey (responded “yes” or “no”). In the instructions, “current task” was defined as what they were doing, what they were intending to do, or what they were supposed to be doing just prior to receiving the survey prompt.

A total of 2185 total survey responses were collected. Responses were then excluded based on response duration (i.e., time taken to complete a survey from start to finish) recorded in seconds, and response latency (i.e., time between when a survey was sent/received and when participants responded) which was categorized based on cut-off times of 15 min, 20 min, 30 min, and 1 hr. Responses were removed if they took more than five minutes to complete (i.e., response duration greater than 300 secs; 47 responses, 2.2%) or if they began more than 30 min after the prompt was sent (481 responses, 22.0%), suggesting that their responses may no longer capture the experiences just prior to the survey. Participants were also excluded if they responded to less than 20% of EMA surveys (i.e., less than 8 responses), suggesting they were not engaged in the study or did not follow instructions. This led to the exclusion of four (6.5%) participants (19 responses, 0.9%). The final sample for the EMA data for Study 1 contained 1638 survey responses from 58 participants with a 70.6% average EMA survey completion rate. The mean survey response duration was 62.85 secs (SD = 37.26 secs; range: 20 to 298 secs). The majority of these responses were completed within 15 min of the probe being sent (83.5%), while an additional 7.4% were within 20 min, and an additional 9.0% were within 30 min.

### Analyses

To examine whether COVID-19 news impacted TUT occurrence measured in daily life, we implemented hierarchical linear regression analyses with mixed models. This involved comparing nested models in terms of model fit, which was determined by comparing Akaike’s information criterion (AIC; Akaike, 1974) and Bayesian information criterion (BIC; Schwarz, 1979)—where lower values indicate better model fit—as well as comparisons of log likelihood statistics using chi-squared analyses.

We first examined the impact of COVID-19 news consumption on TUT occurrence. To test this, the base regression model included a random intercept effect of participants, day-in-study as a fixed effect covariate to control for repeated measures over the course of the study, and COVID-19 news as the fixed effect independent variable of interest, with “not having consumed COVID-19 news” as the reference category. To test whether the relationship between COVID-19 news and TUT occurrence was moderated by motivation, we next added a fixed effect of motivation to the base model, and then tested whether an interaction between motivation and COVID-19 news further improved model fit. If the interaction model improved model fit, we used simple effects analyses to examine how TUT occurrence differed across motivation levels, depending on whether participants consumed COVID-19 news or not.

All analyses were performed using R (version 4.1.0; R Core Team, 2021) in R Studio (version 2021.08.1 + 372; Rstudio Team, 2021), using packages: plyr, dplyr, and tidyr, to organize the data; stats, lme4, lmertest, car, and sjPlot, for modeling and model evaluation; emmeans and effectsize, for simple effects analysis; and viridis and ggplot2, for creating figures. The Tukey method was applied to correct for multiple comparisons in all follow-up simple effects analyses.

## Study 1 results and discussion

**Fig. 1:**
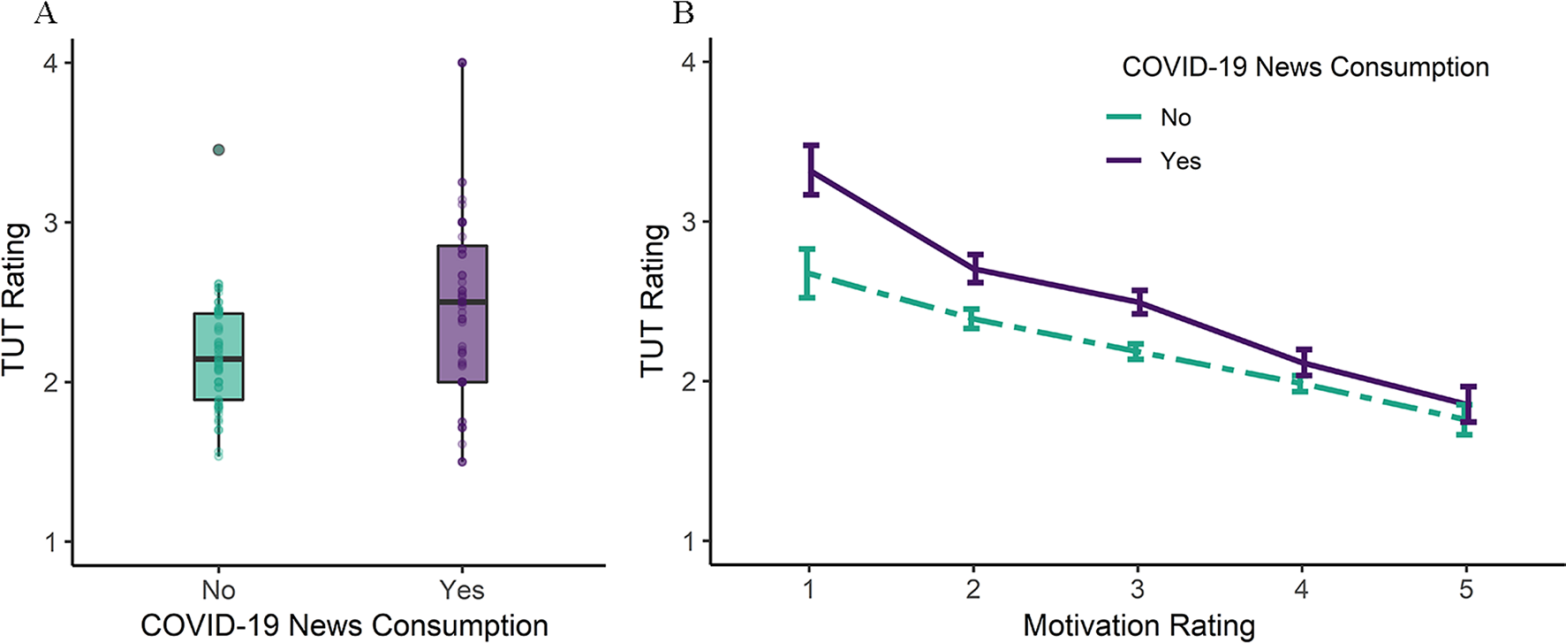
Relationship Between Task-Unrelated Thought Ratings and COVID-19 News Consumption (Study 1). *Note* Task-unrelated thought (TUT) ratings (1 = completely on-task to 4 = completely off-task) are shown as a function of COVID-19 news consumption (panel** A**) and an interaction of motivation ratings and COVID-19 news consumption (panel** B**), from Study 1. Panel A: boxplots of average TUT ratings when participants did and did not consume COVID-19 news; individual averages shown as dots. COVID-19-related news consumption (“Yes”; shown in purple/darker color) was associated with higher ratings of TUT (1 = completely on-task to 4 = completely off-task) compared to the absence of COVID-19-related news consumption (“No”; shown in green/lighter color). Panel** B**: Line graph of average TUT ratings when participants did and did not consume COVID-19 news at different levels of task motivation (1 = not motivated at all to 5 = extremely motivated). Error bars represent the standard error of the mean. The association between TUT and COVID-19 news consumption varied as a function of task motivation

Consistent with our predictions, COVID-19 news consumption was associated with an increase in TUT occurrence (*b* = 0.25, *SE* = 0.06, *χ*^*2*^ (1) = 20.25, *p* < .001), in the base regression model where COVID-19 news was included as the primary independent variable, as depicted in Fig. 1A. Comparing other models to this base model enabled us to establish that any effect of COVID-19 news consumption on TUT occurrence was not solely driven by other variables included in the model. We next added a fixed effect of motivation and then tested the interaction of motivation and COVID-19 news. As reported in Table 1, this interaction was a significant predictor of TUT occurrence (*p* = .008). Further, the inclusion of the interaction improved model fit (*χ*^*2*^ (1) = 7.09, *p* = .008). Descriptive statistics for TUT ratings and Motivation across COVID-19 news consumption are available in Additional file 1: Supplementary Table 5. Details for the model fit comparisons are available in Additional file 1: Supplementary Table 6.

**Table 1:** Linear Regression Model Parameters for Predicting Task-Unrelated Thought from COVID-19 News Consumption (Study 1)

Predictors	*β*	*b*	SE	95% CI [LB, UB]	*Χ*^*2*^ (1)	*p*
COVID-19 news	0.20	0.62	0.16	[0.30, 0.93]	14.30	< .001
Motivation	− 0.23	− 0.22	0.03	[− 0.28, − 0.17]	63.63	< .001
Day	0.03	0.01	0.01	[− 0.00, 0.03]	1.78	.183
COVID-19 news x motivation	− 0.14	− 0.13	0.05	[− 0.23, − 0.03]	7.11	.008

*Note* Rows present the effects of predictors on ratings of TUT (task-unrelated thought, 1 = completely on-task to 4 = completely off-task) from Study 1. Predictors included COVID-19 news (COVID-19-related news consumption, no COVID-19 news consumption as the reference category), task motivation (1 = not motivated at all to 5 = extremely motivated), and day (day-in-study from 1 to 10). The model also included a random effect of participant on the intercept. Analysis of variances was tested through Type III Wald chi-square tests. Model residuals passed assumptions of normality and homogeneity of variance

*β *standardized parameter, *b *unstandardized parameter, *SE *standard error of the estimate, *95% CI *95% confidence interval associated with the unstandardized parameter, *UB *upper bound, *LB *lower bound. *Χ*^*2*^* (1) *chi-squared test statistic associated with comparison between the current model and a null model without the predictor of interest (1 degree of freedom), *p *p-value associated with the chi-squared test statistic

The significant interaction of motivation and COVID-19 news predicting TUT occurrence suggests that task motivation moderates the relationship between COVID-19 news consumption and the occurrence of TUT. Follow-up simple effects analysis demonstrated that when participants had consumed COVID-19 news (compared to when they had not consumed COVID-19 news), the occurrence of TUT was significantly higher, specifically at lower ratings of motivation (1 = “not motivated”, 2 = “somewhat motivated”, 3 = “moderately motivated”; all *p* < .001). When participants were highly motivated to perform their task, COVID-19 news consumption did not impact their TUT ratings (4 = “very motivated”, 5 = “extremely motivated”; all *p* > .200). This relationship is illustrated in Fig. 1B. The details for the simple effects analysis are available in Additional file 1: Supplementary Table 7.

These results support the Motivation and Goal Theory of Current Concerns (Klinger & Cox, 2011), in that watching news related to a concerning current event (i.e., COVID-19) likely brought personal concerns to mind, resulting in increases in TUT when tasks were not immediately related to this concern. However, this raises the question of whether consuming any news media has the same effect. Accordingly, we examined whether the impact of COVID-19 news consumption on TUT occurrence generalizes to the consumption of news in general.

## Study 2 methods

We collected a second dataset from a novel sample for Study 2 to extend our previous finding by examining the consumption of news in general and its link to TUT occurrence. The methods for Study 2 were largely similar to Study 1, in that it included ten days of EMA inquiring about participants’ general news consumption, TUTs, and levels of motivation. However, it did not involve a mindfulness training component or related questionnaires measures. Study 2 was approved by the Conjoint Faculties Research Ethics Board at the University of Calgary.

### Participants

Participants were primarily undergraduate students, recruited through the research participation system at the University of Calgary, and receiving course credit for their participation. Data collection ran from November 18th to December 9th, 2021. During this time, COVID-19 cases were still being reported globally and the omicron variant had just begun to emerge (World Health Organization, 2021), with the first case of the omicron variant in Canada confirmed around November 28th, 2021 (Paas-Lang, 2021). To our best knowledge, active case counts of COVID-19 in Alberta, Canada, did not significantly increase during this time (Canadian Broadcasting Corporation, 2021). Other than the Government of Canada slightly expanding the list of countries with restrictions for entry into Canada on November 30th (Public Health Agency of Canada, 2021), few changes occurred in safety protocols as well. This was also after mask mandates and other restrictions had already been in practice.

Participants were required to be between 17 and 65 years old, fluent in English, and not have practiced mindfulness more than once a week in the past three months. Although the last criterion was not directly relevant to Study 2, we implemented this to closely mimic the protocol from Study 1. Based on a power analysis, with 80% chance of detecting a medium effect size (*d* = 0.50), we aimed to gather valid data from approximately 60 participants. A total of 120 participants signed up for the study, 23 of which dropped before completing informed consent. Five additional participants dropped prior to demographics screening. Fifteen participants were excluded for having recent and regular meditation/mindfulness practice experience. One participant withdrew from the study after beginning their participation. This resulted in a sample of 76 participants. After EMA response-based exclusions (described in more detail below), the final sample was 66 participants. Participants were 19.68 years old on average (SD = 1.73; range: 17 to 25); most identified as female (62, 93.9%), and as white (26, 39.4%) or Asian (28, 42.4%).

### Procedure

Study 2 included 10 days of EMA, as in Study 1. Before starting the EMA, participants completed informed consent and the same EMA instructions and exercise as Study 1, included in Additional file 1: Supplementary Table 4. The question about news consumption was slightly altered to ask about news in general, rather than COVID-19-related news. Participants were then asked to complete the demographics screening survey; however, some participants did not do so until the next day due to technical difficulties. On this second day, participants were sent a practice EMA survey at a random time of day to familiarize them with the methodology, as well as a copy of the instructions to serve as a reminder of the meanings of the EMA questions. Participants then went through the 10 days of EMA starting the day after the practice EMA survey was sent (i.e., day 3).

### Measures–Ecological Momentary Assessment

Other than the question about COVID-19-related news being altered to pertain to news in general, EMA surveys were identical to those in Study 1 to ensure the experience of completing the EMA surveys was as similar as possible between studies, as summarized with the full EMA survey in Appendix Table 3. EMA surveys were sent at the same rate as Study 1 (i.e., 4 times per day within a 10-h time window of the participants’ choice) and participants were instructed to respond when it was possible to do so safely within 15 min of receiving the email prompt. 2459 total complete responses were collected.

Preliminary data checking led to the exclusion of one participant (1.3% of the 76 participants) who gave the same response to every EMA survey, identical to the scenario given in the practice exercise (40 responses, 1.6%). We then applied the same exclusion criteria as in Study 1. For Study 2, both response duration (i.e., time taken to complete a survey from start to finish) and response latency (i.e., time between when a survey was sent/received and when participants responded) were recorded in seconds, allowing for a more precise estimate of average response latency. Responses with a duration of more than five minutes were excluded (i.e., 38 responses, 1.5%). 715 responses (29.1%) were then excluded as they were initiated over 30 min after the surveys were sent, leading to the exclusion of one participant (1.3%) as well. An additional 8 participants (10.5%) with 42 responses (1.7%) were removed for responding to 8 or fewer EMA surveys. The final EMA sample included 1624 responses (66.0%) from 66 participants (86.8%) with a 61.5% average EMA survey completion rate. The mean survey response duration was 57.22 secs (SD = 34.20 secs; range: 15 to 268 secs). The mean response latency was 5.30 min (SD = 6.83 min; range: 0 to 29 min). Similar to Study 1, the majority of responses were within 15 min of the EMA probe being sent out (88.2%), with an additional 4.7% being within 20 min, and an additional 7.1% being within 30 min.

### Analyses

Statistical analyses were identical to Study 1, involving hierarchical linear regression building from a base model. However, rather than examining the impact of COVID-19 news, we examined the impact of consuming news media in general. To approximate the final model from Study 1 while continuing to account for repeated measures, day-in-study was retained as a fixed effect covariate and a random effect of individual was applied to the intercept.

## Study 2 results and discussion

**Fig. 2:**
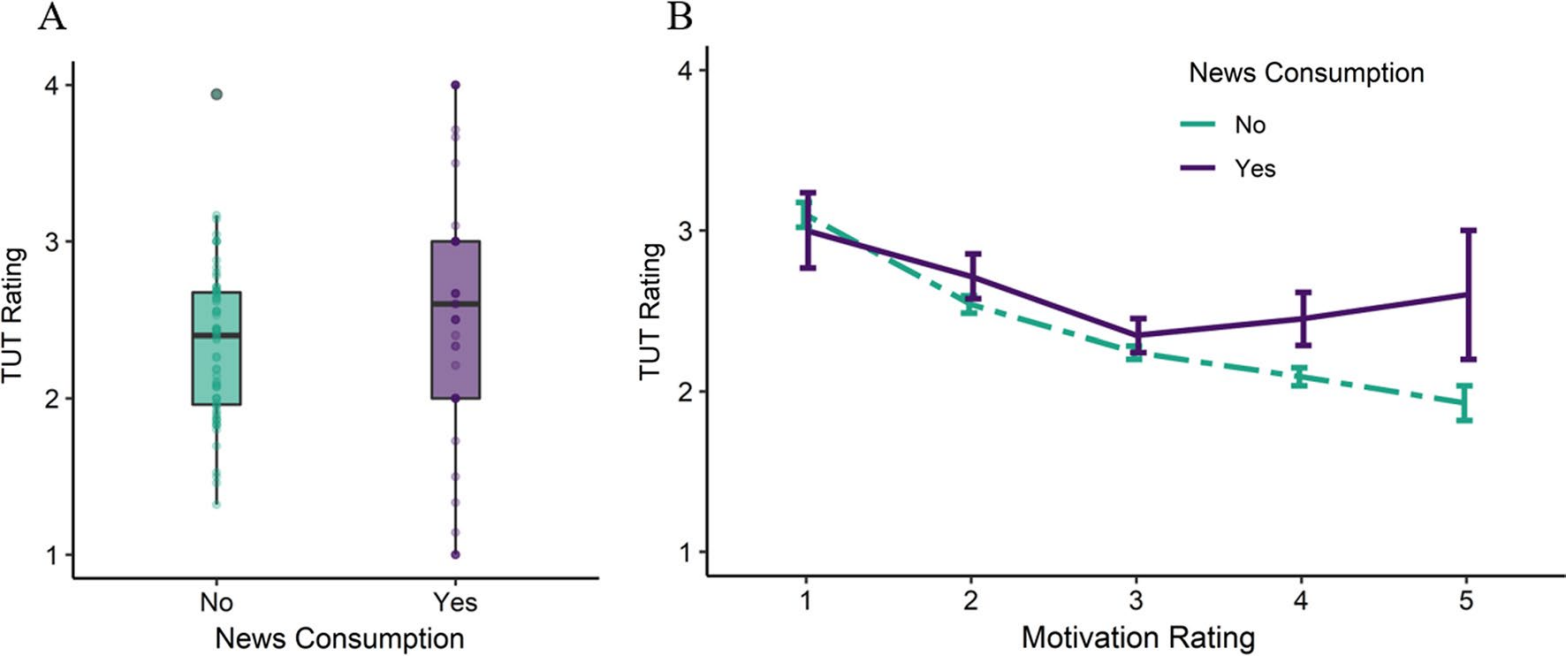
Relationship Between Task-Unrelated Thought Ratings and General News Consumption (Study 2). *Note* Task-unrelated thought (TUT) ratings (1 = completely on-task to 4 = completely off-task) are shown as a function of general news consumption (panel** A**) and an interaction of motivation ratings and general news consumption (panel** B**) which was not significant, from Study 2. Panel** A**: boxplots are of average TUT ratings when participants did and did not consume general news media; individual averages shown as dots. General news consumption (“Yes”; shown in purple/darker color) was associated with higher ratings of TUT compared to the absence of general news consumption (“No”; shown in green/lighter color). Panel** B**: line graph of average TUT ratings when participants did and did not consume general news media at different levels of task motivation (1 = not motivated at all to 5 = extremely motivated). Error bars represent the standard error of the mean. The association between TUT and general news consumption did not significantly vary as a function of task motivation

As a fixed effect independent variable of interest in the base model, general news consumption significantly predicted TUT occurrence (*b* = 0.23, *SE* = 0.08, *χ*^*2*^ (1) = 8.60, *p* = .003). These results are depicted in Fig. 2A. We then examined the interaction effect between general news consumption and task motivation by first adding motivation as a fixed effect, and then adding an interaction of motivation and general news to the model. As an additional fixed main effect, motivation was significant (*p* < .001) as shown in Table 2, and improved model fit (*χ*^*2*^ (1) = 107.69, *p* <0.001), as shown in Additional file 1: Supplementary Table 6. The added interaction of motivation and general news—depicted in Fig. 2B—was not significant, but approached significance (*b* = 0.14, *SE* = 0.07, *χ*^*2*^ (1) = 3.78, *p* = .052), and did not further improve model fit (*χ*^*2*^ (1) = 3.78, *p* = .052). Therefore, we interpreted the results from the model without the interaction of motivation and general news, which tested the main effect of each. Descriptive statistics for TUT and motivation ratings when participants did and did not consume general news are available in Additional file 1: Supplementary Table 5.

**Table 2:** Linear regression model parameters for predicting task-unrelated thought from general news consumption (Study 2)

Predictors	*β*	*b*	SE	95% CI [LB, UB]	*Χ*^2^ (1)	*p*
General news	0.21	0.21	0.08	[0.06, 0.36]	7.82	.005
Motivation	− 0.25	− 0.24	0.02	[− 0.29, − 0.20]	111.09	< .001
Day	− 0.02	− 0.01	0.01	[− 0.02, 0.01]	0.67	.412

*Note* Rows present the effect of predictors on ratings of TUT (task-unrelated thought, 1 = completely on-task to 4 = completely off-task) from Study 2. Predictors included News (general news consumption, no news consumption as the reference category), task motivation (1 = not motivated at all to 5 = extremely motivated), and day (day-in-study from 1 to 10). The model also included a random effect of participant on the intercept. Analysis of variances was tested through Type III Wald chi-square tests. Residuals passed assumptions of normality and homogeneity of variance

*β *standardized parameter, *b *unstandardized parameter, *SE* standard error of the estimate. *95% CI* 95% confidence interval associated with the unstandardized parameter, *UB *upper bound, *LB *lower bound, *Χ*^*2*^* (1)* chi-squared test statistic associated with comparison between the current model and a null model without the predictor of interest (1 degree of freedom), *p p*-value associated with the chi-squared test statistic

The results of Study 2 in terms of the association between general news consumption and TUT occurrence corroborate and extend the COVID-19-related news results from Study 1, such that the consumption of news in general was linked to an increase in TUT. The negative relationship between motivation and TUT occurrence also replicates the results from Study 1. However, the interaction between general news and motivation was not significant, though it approached significance. Together, these two studies suggest that the impacts of news consumption and motivation as fixed predictors are robust, but the interaction between the two is less so.

## General discussion

The goal of these studies was to examine the effects of exposure to salient personal concerns (i.e., COVID-19 news consumption in Study 1, and general news consumption in Study 2) on the occurrence of TUTs. In Study 1, we found that COVID-19-related news consumption was associated with increased TUT occurrence assessed by EMA, particularly when participants were not motivated to do their current task. In Study 2, we demonstrated that the increase in TUT occurrence after exposure to news media was also shown when we considered news in general. Although the lower levels of motivation also predicted higher levels of TUT in both studies, the interaction of motivation and news in general was not significant in Study 2. Taken together, these findings suggest that news media may cue current concerns thereby increasing TUT occurrence, whether or not the focus of the news is on a specific concerning current event.

Our findings of a positive relationship between COVID-19-related news consumption as well as general news consumption and TUT occurrence supports the Motivation Theory of Current Concerns (Klinger & Cox, 2011). Given that TUTs tend to focus on current concerns (e.g., Geerligs, 1995; Smallwood et al., 2009), it is plausible that exposure to news media increased the salience of current concerns in both samples. In turn, this distracted participants from their ongoing tasks, thereby increasing TUT occurrence. Our results are consistent with lab-based reports of increased TUT occurrence following exposure to fake news reports (Antrobus et al., 1966) or cues related to current concerns (e.g., Kopp et al., 2015; McVay & Kane, 2013). Importantly we extend these findings demonstrating a relationship between current concerns and TUTs beyond the lab setting into everyday life. We also extend recent findings reporting that consumption of media in general during the COVID-19 pandemic changes thought patterns (McKeown et al., 2021). Our results additionally complement recent work showing that people who are worried about the pandemic report higher rates of TUTs, including TUTs specifically related to COVID-19 (Jun et al., 2021).

In Study 1, we also found that COVID-19 news consumption positively correlated with TUT occurrence at lower to middle levels of motivation. This is consistent with past studies reporting that motivation is a key predictor of TUT rates (e.g., Robison & Unsworth, 2018; Seli et al., 2015, 2016). Specifically, when people are less motivated to do their tasks, they tend to engage in TUTs, allowing themselves to think about task-unrelated topics including their current concerns. In contrast, when people are highly motivated to complete their tasks, they usually show lower rates of TUTs. Seli and colleagues (2016) speculate that this is because we are better able to detect or prevent TUTs when we are highly motivated toward a current task. This may explain why COVID-19-related news consumption did not modulate TUT occurrence when our participants were highly motivated toward their tasks: they may have been better able to prevent TUTs regardless of the salience of task-unrelated current concerns. However, the interaction between motivation and general news consumption only approached significance in Study 2, suggesting this interaction effect is not robust. This may have be a result of differences in characteristics between our two samples, as well as differences between COVID-19 news and news in general, highlighting potential limitations to these studies.

### Limitations and future directions

Recruiting a new group of individuals for Study 2 allowed us to establish that the main effect of COVID-19 news consumption on TUT occurrence found in Study 1, generalizes to any news consumption in a different sample. However, the differences between the samples may have also contributed to the differences in results regarding the interaction between news and motivation observed between these two studies and must therefore be taken into consideration.

Although both samples were rather limited in terms of diversity—being convenience samples primarily composed of white or Asian females in North America—they differed in terms of age and education. The sample from Study 1 was primarily recruited from graduate programs and local communities, having a broad age range from 19 to 63 years, while the sample for Study 2 was composed of individuals currently enrolled in an undergraduate program between the ages of 17 and 25. Age has previously been shown to impact TUTs in daily life (Maillet et al., 2018). It is also likely that younger and older adults differ in terms of their concerns. For example, risk of hospitalization and death from COVID-19 varies by age group (Centers for Disease Control and Prevention, 2022). There are likely additional differences in terms of work/school demands which could influence the nature of their concerns. Aside from age differences, COVID-19 specific concerns and news consumption patterns may have varied across samples, given that the data for Study 2 were gathered over one year after Study 1, long after changes to daily life due to the pandemic had been established.

The most notable difference across the samples in Study 1 and Study 2 is that half of the participants in Study 1 were enrolled in a mindfulness training program. Although our control analyses suggested that mindfulness training did not have an impact on TUT occurrence, it is possible that enrollment in a study that involves a daily practice of mindfulness influences one’s motivation levels and awareness of their attention (regardless of whether they were assigned to the mindfulness training or waitlist control group). Another potential difference between the two studies that may contribute to differences in results lies in the content of news. While Study 1 only focused on COVID-19 news, which was presumably concerning during both time periods, Study 2’s focus on general news may have included some more positive topics when it came to areas such as popular culture and sports. However, this seems to be an unlikely explanation because the effect sizes of COVID-19 news and general news were similar when regression models included motivation (COVID-19 news *β* = 0.20 and general news *β* = 0.21; see Tables 1 and 2). Moreover, news media has a tendency to focus more on negative topics (Leonhardt, 2021), so even though Study 2 examined general news, it seems likely that there was a negative bias in news content. Together, these differences between our samples may have contributed to the observed differences in how motivation interacts with news consumption in predicting the occurrence of TUT across our two studies. Critically, the impact of news on TUT occurrence was robust against these differences in sample characteristics.

Another important point of consideration is the validity of experience sampling methods, including EMA. While experience sampling is the most common method of measuring TUTs (see Smallwood & Schooler, 2006, 2015; for review), the frequency at which participants report TUT occurrence has previously been shown to vary by sampling rate (Seli et al., 2013) and the wording of sampling probes (Weinstein et al., 2018). Although the frequency of TUT reports fluctuates, other studies have shown that self-report of TUTs correspond with consistent effects on performance, sensitivity to stimuli, and functional brain activity (see Smallwood & Schooler, 2015, for review; Fox et al., 2015). TUT occurrence rates from in-lab experience sampling have also been correlated with TUT occurrence measured via EMA in daily life (McVay et al., 2009).

A recent report from Kane et al., (2021) reviewed potential confounds to experience sampling and demonstrated that the reliability and validity of experience sampling reports partly depended on how probes asked about TUTs in laboratory settings. In short, they found that responses to probes similar to those used in the current studies could be influenced by participants’ reactions to their performance on in-lab tasks and may be confounded with participants’ confidence in their probe responses. Similarly, participants’ responses to the EMA surveys in the current studies may be influenced by question order. Though some experience sampling studies present questions related to participants’ thoughts first (e.g., Kane et al., 2017), our survey started with questions related to the current task, before asking about thoughts. Given the often ephemeral quality of thoughts, having questions related to thoughts presented after other questions in EMA probes may impact participants’ responses. Importantly, these questions about the task were very brief and—given participants’ familiarity with the surveys after training—should only have taken a few seconds to answer. Participants were also repeatedly told that they would be asked about their thoughts just before receiving the probes. While it is still possible for items related to the task to influence subsequent responses to items related to TUTs, this would unlikely have had a strong effect in the current studies. As little research has examined the impact of question order in EMA studies, future studies may counterbalance question order to examine their impact on participants’ responses. Finally, as with most Likert-style response scales, the extreme options of the response scale to the probes used in the current studies may have also had different interpretations across participants.

While there are certainly issues with validity and reliability of experience sampling probes for measuring TUT, our studies included several key features in the methods to mitigate these effects. First, our studies involved a set of instructions consistent with those from other studies (e.g., Kane et al., 2017; McVay et al., 2009; Mills et al., 2018), describing all parts of the EMA surveys as shown in Additional file 1: Supplementary Table 4. The instructions repeatedly reminded participants that they were to respond to the EMA surveys within a short time interval (when safe to do so) and that they were to characterize their thoughts occurring just before receiving the survey. The instructions also described each survey question in detail, and the meanings of response options including those on Likert scales. Participants had to respond to the instructions survey, stating that they understood each question, before moving on. Second, our TUT-focused question was on a four-point scale to remove mid-point responses and require participants to state their thoughts as more off-task or on-task. This reduced the potential for ambiguous responses. Third, participants were required to complete an EMA survey exercise, according to a specific scenario provided to them, in order to proceed in the study. Finally, they were sent a practice EMA survey to fill out as if it were one of their daily surveys prior to the start of the EMA portion of the study, to familiarize them with the EMA procedure.

Besides EMA, there is no standard method of assessing TUT occurrence during everyday life and no standard indirect measure of TUT, making experience sampling a necessity. This comes with the limitations of observational research; that is, we cannot determine the causal direction of the relationship between news consumption and TUT frequency. Since our EMA survey required participants to report recent momentary TUTs, but news consumption within two hours of the EMA, it is conceivable that the news consumption preceded TUT occurrence. However, we cannot rule out the possibility that participants may have been prompted to view the news because they were experiencing TUTs related to news topics—including COVID-19—earlier, or because they were not focused on any particular task. While our results show a robust relationship between news and TUT occurrence, and this corroborates theories on current concerns, further research is needed to determine the causal nature of this relationship. Additionally, the data collection periods for our studies were limited to a few weeks, not allowing us to examine whether effects are shown consistently over longer periods of time. While we have shown that the relationship between news consumption and TUT occurrence was present in 2020 and 2021, we cannot definitively state whether the observed patterns existed before the COVID-19 pandemic or will continue to exist. However, given the consistency of our results in terms of news, we speculate that the relationship between news consumption and TUT occurrence reported here will persist over time and extend to other specific news topics.

## Conclusions

The goal of these studies was to explore factors related to current concerns that modulate TUT occurrence in everyday life. These findings inform us on potential avenues to gain better control over TUT occurrence in order to reduce the detrimental effects of TUTs. Our results demonstrated that consuming news related to COVID-19 and news in general was associated with higher TUT occurrence in daily life, and that the impact of news on TUTs may vary as a function of motivation toward current tasks. This will require more research to determine the causal direction of the relationship between news and TUT occurrence, as well as to explore how and when task motivation may moderate this relationship.

The implications of these results are two-fold. In terms of methodological contributions, these results emphasize the importance of considering daily life experiences when studying TUTs in ecological settings. In terms of practical implications, they caution us to be more considerate of when we view the news or engage in other daily activities that can significantly impact the occurrence of TUTs. For instance, we may limit our consumption of news (Lades et al., 2020) or restrict it to times when TUTs will not disrupt more important tasks. News providers may also re-consider how they are presenting stories (De Hoog & Verboon, 2020). For instance, journalists could apply a Constructive Journalism approach (see, MacDonald, 2021; for review) to present solutions to current issues or actions individuals can take to mitigate their concerns. Taken together, being more cognizant of factors that influence our attentional focus is an important step toward improving our ability to focus on the task-at-hand as desired.

### Supplementary Information

**Additional file 1.** Suuplemantary tables.

## Data Availability

The data used for this study are available from the corresponding author on request.

## Appendix: Ecological momentary assessment survey

see Table 3.

**Table 3:** Ecological momentary assessment survey questions

Survey question	Response options
What is your current task?	(Fill in response)
How easy or difficult was this task?	Extremely easy Somewhat easy Moderate Somewhat difficult Extremely difficult
How interesting did you find the task?	Not interesting at all Somewhat interesting Moderately interesting Very interesting Extremely interesting
How motivated were you to do the task?	Not motivated at all Somewhat motivated Moderately motivated Very motivated Extremely motivated
How on-task (task-related) or off-task (task-unrelated) was your thought?	Completely on-task Somewhat on-task Somewhat off-task Extremely off-task
Please check all the topics that your thought involved. ^a^	Pandemic-related health concerns of yourself or loved ones Uncertainty about pandemic-related financial/job concerns Concerns about way of life after pandemic Other pandemic-related concerns Concrete steps toward achieving a goal Fantastical musings not grounded in reality Other
Did your thought occur intentionally (under your control) or unintentionally (outside of your control)?	Unintentionally Intentionally
How aware were you of the contents of your thought?	Not aware at all Somewhat unaware Moderately aware Very aware Extremely aware
Was your thought oriented toward the external environment, your inner world, or a bodily sensation?	External environment Inner world Bodily sensations
Where in time was your thought focused?	Past Present Future No particular time
Was your mind freely moving from one thought to another, or focused on a specific topic? This question pertains to your thoughts occurring in the past few minutes	Very focused on one topic Somewhat focused on one topic Somewhat freely moving from one thought to another Very much freely moving from one thought to another Extremely freely moving from one thought to another
How positive or negative do you feel at this moment?	Extremely negative Somewhat negative Neutral Somewhat positive Extremely positive
Have you read, listened to, or watched (COVID-19-related news/the news) in the last two hours? ^b^	Yes No
How did the news make you feel? (Reverse coded.) ^c^	Positive Neutral Negative
What was the news about? ^c^	COVID-19-related topics Other health-related topics Natural disaster Politics Economy Sports Popular culture Other
Where did you obtain the news? ^c^	Newsprint/website Radio Television/Online Video Social Media

*Note* In the current study, we focused only on the items that were relevant to our primary research question: task-unrelated thought ratings (question 5a), news consumption (question 12a), and motivation ratings (question 4). The other questions were intended to address different research questions that are not relevant to the current study. For instance, in a previous paper (Kam et al., 2021), we examined affective valence. The remaining items on the survey were used for piloting purposes and were not included in the analyses. Responses are numbered for questions where responses were considered on a Likert scale. For each ecological momentary assessment probe, questions and responses were presented to participants in the same order shown, but without numerical coding for the responses

^a^Participants were only asked about the topics of their thoughts if they rated their thoughts as somewhat or extremely off-task

^b^For Study 1, this question asked about COVID-19-related news. For Study 2, this question asked about news in general

^c^These questions were only presented in Study 2

## Abbreviations

TUT: Task-unrelated thoughts
COVID-19: Coronavirus disease
EMA: Ecological momentary assessment
AIC: Akaike’s information criterion
BIC: Bayesian information criterion
SE: Standard error
CI: Confidence interval
UB: Upper bound
LB: Lower bound
df: Degrees of freedom

## Footnotes

1 The original demographics questionnaire used the term “Caucasian”. As outlined in the American Psychological Association Inclusive Language guidelines (2021), this term is not an appropriate alternative to “white” or “European” and should not be used.
