# Supplementary material for: Task-unrelated thought increases after consumption of COVID-19 and general news
Source: Cogn Res Princ Implic. 2022 Jul 25;7:69. doi: 10.1186/s41235-022-00420-7 (PMC9309453; doi:10.1186/s41235-022-00420-7)
Supplement: Supplementary file 1 — Additional file 1. Suuplemantary tables. [file 41235_2022_420_MOESM1_ESM.docx]

**Task-Unrelated Thought Increases after Consumption of COVID-19 and General News**

**Hart, C. M., Mills, C., Thiemann, R. F., Andrews-Hanna, J. R., Tomfohr-Madsen, L., & Kam, J. W. Y.**

**Supplementary Materials**

**Supplementary Table 1**

***Linear Regression Model Parameters for Predicting Task-Unrelated Thought by Mindfulness Training Group (Study 1)***

| Predictors | *β* | *b* | *SE* | 95% CI  [LB, UB] | *Χ^2^* (1) | *p* |
| --- | --- | --- | --- | --- | --- | --- |
| Mindfulness Training Group | -0.01 | -0.01 | 0.08 | [-0.17, 0.15] | 0.02 | .882 |
| Day | 0.03 | 0.01 | 0.01 | [-0.01, 0.03] | 1.63 | .202 |

*Note*. Values represent the results of a linear mixed effects regression model, predicting ratings of task-unrelated thought (1 = completely on-task to 4 = completely off-task) as assessed via ecological momentary assessments over a 10-day period during which the mindfulness training group were asked to practice mindfulness daily. Predictors included Mindfulness Training Group (26 waitlist controls vs 32 mindfulness training, waitlist controls as reference group) and day (day-in-study from 1 to 10), as well as a random effect of participant on the intercept. Models were tested through Type III Wald chi-square tests. Residuals from this model did not pass the assumption of normality (Shapiro-Wilk’s test *W* = 0.96, *p* < .001).

These results indicate that mindfulness training did not significantly predict the occurrence of task-unrelated thoughts (TUT) during the 10 days of Study 1. This is in contrast to other research, reviewed in previous meta-analyses (Gill et al., 2020; Yakobi et al., 2021). The literature so far demonstrates some cognitive functioning improvements after mindfulness training which may connect to TUT occurrence. However, results are mixed, and more research is required to address inconsistencies across studies.

Gill, L. N., Renault, R., Campbell, E., Rainville, P., & Khoury, B. (2020). Mindfulness induction and cognition: A systematic review and meta-analysis. *Consciousness and Cognition*, *84*, 102991. <https://doi.org/10.1016/j.concog.2020.102991>

Yakobi, O., Smilek, D., & Danckert, J. (2021). The effects of mindfulness meditation on attention, executive control and working memory in healthy adults: A meta-analysis of randomized controlled trials. *Cognitive Therapy and Research*, *45*(4), 543-560. <https://doi.org/10.1007/s10608-020-10177-2>.

**Supplementary Table 2**

***Descriptive Statistics and Group Comparisons of Questionnaire Scores at Baseline Assessment (Study 1)***

| Questionnaire | Mindfulness Training Mean (SD) | Waitlist Control Mean (SD) | *t* (60) | 95% CI  [LB, UB] | *p* |
| --- | --- | --- | --- | --- | --- |
| MWQ | 3.91 (0.90) | 4.10 (0.92) | 0.81 | [-0.27, 0.65] | .421 |
| MW-D | 4.46 (1.29) | 3.71 (1.61) | -2.04 | [-1.48, -0.01] | .046 |
| MW-S | 4.50 (1.05) | 4.33 (1.37) | -0.57 | [-0.79, 0.44] | .574 |

*Note.* Differences between a mindfulness training group and a waitlist control group were examined via independent samples t-tests.

MWQ = mind wandering questionnaire; scores ranged from 1 to 6 (1 = “almost never” to 6 = “almost always”). MW-D = mind wandering deliberate subscale; scores ranged from 1 to 7 (1 = “rarely” / “not at all true” / “almost never”, to 7 = “a lot” / “very true” / “almost always”). MW-S = mind wandering spontaneous subscale; scores ranged from 1 to 7 (1 = “rarely” / “not at all true” / “almost never”, to 7 = “a lot” / “very true” / “almost always”). 95% CI = 95% confidence interval of the difference between groups, UB = upper bound. LB = lower bound.

While these analyses suggested significant group differences on the MW-D, examining the post-training scoring differences showed that when accounting for this baseline difference, group was not a significant predictor of MW-D post-training (Supplementary Table 3). This suggests that the group difference on MW-D was constant from pre- to post-training and was therefore not impacted by mindfulness training.

**Supplementary Table 3**

***Linear Regression Models for Post-Training Questionnaire Scores by Mindfulness Training Group (Study 1)***

| Variable | *β* | *b* | *SE* | 95% CI  [LB, UB] | *F* (2,59) | *p* | *R*^2^_Adj_ |
| --- | --- | --- | --- | --- | --- | --- | --- |
| MWQ Post-Training | | | | | 30.12 | < .001 | .49 |
| Group | -0.08 | -0.07 | 0.16 | [-0.38, 0.25] |  | .679 |  |
| MWQ Baseline | 0.71 | 0.67 | 0.09 | [0.49, 0.84] |  | < .001 |  |
| MW-D Post-Training | | | | | 31.97 | < .001 | .50 |
| Group | 0.16 | 0.23 | 0.26 | [-0.28, 0.74] |  | .377 |  |
| MW-D Baseline | 0.70 | 0.64 | 0.09 | [0.47, 0.82] |  | < .001 |  |
| MW-S Post-Training | | | | | 15.51 | < .001 | .32 |
| Group | 0.05 | 0.05 | 0.23 | [-0.42, 0.52] |  | .827 |  |
| MW-S Baseline | 0.58 | 0.54 | 0.10 | [0.34, 0.73] |  | < .001 |  |

*Note.* Each set of rows presents two independent variables’ effects on one of three post-training questionnaires from three separate linear regression models. Group refers to mindfulness training group and waitlist controls, with waitlist controls as the reference group.
MWQ = mind wandering questionnaire; scores ranged from 1 to 6 (1 = “almost never” to 6 = “almost always”). MW-D = mind wandering deliberate subscale; scores ranged from 1 to 7 (1 = “rarely” / “not at all true” / “almost never”, to 7 = “a lot” / “very true” / “almost always”). MW-S = mind wandering spontaneous subscale; scores ranged from 1 to 7 (1 = “rarely” / “not at all true” / “almost never”, to 7 = “a lot” / “very true” / “almost always”). *β* = standardized parameter. *b* = unstandardized parameter. *SE* = standard error of the estimate. 95% CI = 95% confidence interval associated with the unstandardized parameter. UB = upper bound. LB = lower bound. *R*^2^_Adj_ **=** adjusted R squared.

The mindfulness training group and waitlist control group did not significantly differ on questionnaire measures of TUT after the training period while controlling for group differences at baseline (Supplementary Table 2).

**Questionnaire Measures**

The complete list of questionnaires implemented in the original study is described below in administration order. They can be largely categorized as attention questionnaires relevant to the supplementary results reported above (Supplementary Tables 2 and 3, as part of Study 1), well-being questionnaires relevant to a previously published paper on this data set (Kam et al., 2021), and questionnaires for other purposes. We implemented questionnaires assessing tendencies to engage in task-unrelated thoughts before and after the mindfulness training. Instead of assessing trait level task-unrelated attention, the goal was to assess tendencies to engage in task-unrelated thoughts in everyday life over the past two weeks. Therefore, questionnaire wording was altered slightly at baseline and post-study assessment periods to ask participants to respond based on their tendencies over the “past two weeks”. Our intention was to determine whether a retrospective measure (questionnaire) and a momentary measure (EMA) would provide unique information for task-unrelated attention.

- Attention (Task-Unrelated Thought/Mind Wandering):
  - The Mind Wandering Questionnaire

Mrazek, M. D., Phillips, D. T., Franklin, M. S., Broadway, J. M., & Schooler, J. W. (2013). Young and restless: Validation of the Mind-Wandering Questionnaire (MWQ) reveals disruptive impact of mind-wandering for youth. *Frontiers in Psychology*, *4*, 560. <https://doi.org/10.3389/fpsyg.2013.00560>

- - The Mind Wandering Deliberate and Spontaneous Scales

Carriere, J. S., Seli, P., & Smilek, D. (2013). Wandering in both mind and body: Individual differences in mind wandering and inattention predict fidgeting. *Canadian Journal of Experimental Psychology/Revue Canadienne de Psychologie Expérimentale*, *67*(1), 19. [https://doi.org/10.1037/a0031438](https://psycnet.apa.org/doi/10.1037/a0031438)

- Dispositional Mindfulness:
  - The Mindful Attention and Awareness Scale

Brown, K. W., & Ryan, R. M. (2003). The benefits of being present: mindfulness and its role in psychological well-being. *Journal of Personality and Social Psychology*, *84*(4), 822–848. <https://doi.org/10.1037/0022-3514.84.4.822>

- Emotional Regulation:
  - The Emotional Regulation Questionnaire

Gross, J.J., & John, O.P. (2003). Individual differences in two emotion regulation processes: Implications for affect, relationships, and well-being. *Journal of Personality and Social Psychology*, *85*(2), 348-362. [https://doi.org/10.1037/0022-3514.85.2.348](https://psycnet.apa.org/doi/10.1037/0022-3514.85.2.348)

- Current Affective Experiences:
  - The Positive Affect and Negative Affect Scale (Watson et al., 1988)

Watson, D., Clark, L. A., & Tellegen, A. (1988). Development and validation of brief measures of positive and negative affect: The PANAS scales. *Journal of Personality and Social Psychology*, *54*, 1063–1070. <https://doi.org/10.1037//0022-3514.54.6.1063>

- The Patient Reported Outcomes Measurement Information System:

Cella, D., Riley, W., Stone, A., Rothrock, N., Reeve, B., Yount, S., Amtmann, D., Bode, R., Buysse, D., Choi, S., Cook, K., Devellis, R., Dewalt, D., Fries, J. F., Gershon, R., Hahn, E. A., Lai, J. S., Pilkonis, P., Revicki, D., Rose, M., Weinfurt, K., Hays, R. (2010). The patient reported outcomes measurement information system (PROMIS) developed and tested its first wave of adult self-reported health outcome item banks: 2005-2008. *Journal of Clinical Epidemiology*, *63*(11), 1179–1194. <https://doi.org/10.1016/j.jclinepi.2010.04.011>

- - Depression Scale

Pilkonis, P. A., Yu, L., Dodds, N. E., Johnston, K. L., Maihoefer, C. C., & Lawrence, S. M. (2014). Validation of the depression item bank from the Patient-Reported Outcomes Measurement Information System (PROMIS) in a three-month observational study. *Journal of Psychiatric Research*, *56*, 112–119. <https://doi.org/10.1016/j.jpsychires.2014.05.010>

- - Anxiety Scale

Pilkonis, P. A., Choi, S. W., Reise, S. P., Stover, A. M., Riley, W. T., & Cella, D. (2011). Item banks for measuring emotional distress from the Patient-Reported Outcomes Measurement Information System (PROMIS): Depression, anxiety, and anger. *Assessment*, *18*, 263–283. [https://doi.org/10.1177/1073191111411667](https://doi.org/10.1177%2F1073191111411667)

- - Sleep Disturbance Scale
- Self Compassion (self-judgement, isolation, and over-identification):
  - The Self-Compassion Scale

Neff, K. D. (2003). Development and validation of a scale to measure self-compassion. Self and Identity, *2*(3), 223-250. <https://doi.org/10.1080/15298860309027>

**Supplementary Table 4**

***Instructions for Ecological Momentary Assessment Survey***

| Question/Item | Instructions |
| --- | --- |
| 1. What is your current task? | Here, we would like you to describe what you were just doing – i.e. your current “task”. Your current task need not be work related. Instead, it is simply what you are supposed to be or intended to be doing at the moment.  For example, your current or intended “task” could be any of the following:  * working in your home office  * grocery shopping  * watching a movie  * strolling in the park  * having dinner  * reminiscing about your last vacation  * idling on the couch |
| 1. How easy or difficult was the task? | Please rate how easy or difficult you find your current task to be at the moment. The difficulty can be either mental or physical.  For example: * you may respond 1 = extremely easy, if you were idling on the couch. * you may respond 2 = somewhat easy, if you were taking a stroll in the park. * you may respond 5 = extremely difficult, if you were performing a new task at work. |
| 1. How interesting did you find your task? | Please rate how interesting you find your current task to be at the moment. For example:  * you may respond 1 = not interesting at all, if you were grocery shopping.  * you may respond 4 = very interesting, if you were watching a movie.  * you may respond 5 = extremely interesting, if you were reminiscing about your last vacation. |
| 1. How motivated were you to perform this task? | Please rate how motivated you were to perform the current task at the moment.  For example:  * you may respond 1 = not motivated at all, to run in the rain.  * you may respond 3 = moderately motivated, to do laundry because you are out of socks.  * you may respond 5 = extremely motivated, to complete a task at work. |
| - 1. How on task (task-related) or off task (task-unrelated) were your thoughts? | Here, we ask that you rate how focused your thoughts were on your current task.  For example:  * you would respond 1 = completely on task, if you were completely focused on performing a work-related task.  * you would respond 2 = somewhat on task, if while going for a run you were generally paying attention to the path ahead of you but occasionally thinking about what to have for lunch after the run.  * you would respond 3 = somewhat off task, if you were ruminating about a family issue while watching a movie and not paying attention to the movie at all.  * you would also respond 4 = extremely off task if you were surfing the web on your phone while you were supposed to be studying for an exam. ^a^ |
| - 1. If you responded “off task” to the above question, we would then ask you to describe what your off task thought was about. | We ask that you report what your off task or task-unrelated thought was focused on.  For example:  * you would respond 1 = pandemic-related health concerns, if you were thinking about how to minimize the chance that you or your parents would get COVID 19.  * you would respond 2 = pandemic-related financial or job concerns, if you were thoughts were focused on whether you have enough money to pay rent next month.  * you would respond 3 = concerns about way of life after pandemic, if you were thinking about whether your favourite restaurant will continue to have limited seating options.  * you would respond 4 = concrete steps to achieve a goal, if you were thoughts were focused on what you need to achieve today to make progress on your project.  * you would respond 5 = fantastical musings not grounded in reality, if you were thinking about what life is like on Mars, or about how awesome it would be to make the pandemic magically go away.  * you would respond 6 = other, if the above categories do not accurately describe your thoughts. |
| 1. Did your thoughts occur intentionally or unintentionally? | We would like you to describe whether your thoughts occurred intentionally (i.e., on purpose and under your deliberate control), or unintentionally (i.e., spontaneously and outside of your control).  For example:  * you would respond 1 = unintentionally, if you somehow spontaneously started to think about your last vacation while you were working.  * you would respond 2 = intentionally, if you stayed focused on watching a movie on purpose, or if you stopped focusing on a movie and under your deliberate control you started to think about your friend who will be visiting next summer. |
| 1. How aware were you of what you were just thinking about? | Please rate how aware you were of your ongoing thoughts. This question is specifically asking about whether you are aware of the content of your thoughts, and not whether you are aware that your thoughts are related to the ongoing task.  For example:  * you would respond 1 = not aware at all, if the email notification caught you by surprise and only then did you realize you had been reminiscing about a concert.  * you would respond 5 = extremely aware, if you are fully aware that you were thinking about the concert last weekend while you are working.  Note in this case that you need not be aware that your thoughts have drifted away from work. All that matters for this question is that you are aware of the content of your thoughts. |
| 1. Were your thoughts about the external world, your internal world, or about bodily sensations? | We would like you to state whether your thoughts were about the external world (e.g. any one, thing, or place beyond your body at the moment), about your internal world (e.g. anything going on in your head, including your thoughts and memories), or about a bodily sensation you were experiencing (e.g. your breathing, an itch on your leg, or a headache).  For example:  * you would respond 1 = external environment, if you were focused on the loud drilling noise from a nearby construction site.  * you would respond 2 = inner world, if you were reminiscing about last summer.  * you would respond 3 = bodily sensations, if your thoughts were focused on the mosquito bite on your leg. |
| 1. Where in time were your thoughts focused? | We ask that you report whether your thoughts were focused on a particular point in time, and if so whether they were focused on the past, present, or future.  For example:  * you would respond 1 = past, if you were thinking about the movie you watched yesterday night.  * you would respond 2 = present, if your thoughts were focused on the ongoing loud drilling noise.  * you would respond 3 = future, if you were thinking about planning a party for your friend’s birthday.  * you would respond 4 = no particular time, if you were singing a song stuck in your head. |
| 1. Was your mind wandering around freely moving from one thought to another (in the past two minutes)? | Here, we ask that you rate how much your thoughts have been moving from one topic to another. Thoughts tend to move freely when there is no overarching purpose or direction to your thinking. We would like you to capture how much your thoughts drifted from one thing to another, without focusing on any one thing for too long. We understand that your thoughts may be drifting around for the past few minutes, but at the specific moment you received the email notification, you may be only thinking of one topic. So for answering this question, please think back to the past two minutes.  For example:  * you would respond 1 = very focused on one topic, if in the span of the few minutes, you stay focused on creating a grocery list, containing all items you need for the week. Here your thoughts were focused on one topic for an extended period of time.  * you would respond 5 = extremely freely moving from one thought to another, if in the span of the few minutes, you imagine yourself having dinner this evening, then wonder if you've been eating much fast food recently, then notice a smudge on the computer screen, then remember that you have to clean the bathroom when you get home*.* In this case, your mind was wandering around freely and quickly jumping from one thought to another. |
| 1. How positive or negative do you feel at the moment? | Here, we ask that you rate the valence of your thoughts - specifically whether it is positive or negative. Positive valenced feelings can include being happy, excited, relaxed, calm, and content among others. Negative valenced feelings can include being anxious, stressed, afraid, sad, and worried among others.  For example:  * you would respond 1 = extremely negative, if you felt worried about your sister who works in the emergency room.  * you would respond 3 = neutral, if you were thinking about what movie to watch tonight.  * you would respond 5 = extremely positive, if you were excited at the thought of making your first sourdough bread this weekend. |
| - 1. Have you read, listened to, or watched (COVID-19 related news/the news) in the past two hours? ^b^ | This question has nothing to do with your thoughts *in the moment.* We simply would like to know if you have come across (COVID-19 related news/any news) media in the past two hours.  For example:  * you would respond 1 = yes, if you spent the last 30 minutes (or two hours) watching (COVID-19 related news/the news).  * you would respond 2 = no, if you have not read, listened to or watched (COVID-19 related news/the news) in the past two hours. |
| - 1. If you had read/listened to/watched the news, we would then ask you how the news made you feel. ^c^ | This question has nothing to do with your thoughts in the moment. We would like to know how the news made you feel at the time. The news may make you feel positively if it's about something that you felt was beneficial or exciting for yourself or others, while it may have a negative impact on your emotions if it's related to something harmful or disappointing.  For example:  * you would respond 1 = positive, if the news was about the opening of a new recreation centre in your area that you were excited about.  * you would respond 2 = neutral, if the news was on a mild weather forecast which didn't make you feel particularly positive or negative.  * you would respond 3 = negative, if the news was about the loss of an endangered species which made you sad. |
| - 1. If you had read/listened to/watched the news, we would then ask you what the news story you spent the most time reading/listening to/watching was about. ^c^ | This question has nothing to do with your thoughts in the moment. We would like to know what the news you read, watched, or listened to was most focused on.  For example:  * you would respond 1 = COVID-19 related topics, if you were watching a news story that was focused on the efficacy of COVID-19 vaccines.  * you would respond 2 = other health-related topics, if the news was on topics other than COVID-19, but still related to health, such as a new treatment for anxiety.  * you would respond 3 = natural disaster, if the news you spent the most time reading about in the past two hours was about an earth quake in the pacific ocean.  * you would respond 4 = politics, if the news was about a new law related to littering.  * you would respond 5 = economy, if the news was on a change in the value of gold.  * you would respond 6 = sports, if the news was about a hockey player scoring their first goal on a new team.  * you would respond 7 = popular culture, if the news was mainly discussing a celebrity divorce.  * you would respond 8 = other, if none of the previous areas described what the news story you spent the most time on in the past two hours was about. |
| - 1. If you had read/listened to/watched the news, we would then ask you where you obtained the news. ^c^ | This question has nothing to do with your thoughts in the moment. We would like to know where you obtained the news you were reading, watching, or listening to.  For example:  * you would respond 1 = news print/website, if the news came directly from an article in the newspaper or on a news website such as CBC news.  * you would respond 2 = radio, if you heard the news on a radio channel.  * you would respond 3 = television/online video, if the news came from the televised news or a news video online.  * you would respond 4 = social media, if the news came from written or video posts on social media sites such as twitter or tiktok. |
| Transition | To help you understand better how to respond to the above questions, let’s do an exercise! Below, we have a hypothetical situation you may find yourself in when you receive our email. Please read the description of the hypothetical situation and respond to each of the questions in the survey. |
| Exercise ^d^ | Imagine you are preparing your dinner while listening to the radio. You are using a particularly involved recipe, requiring you to be careful to follow it and ensure you have all of the ingredients ready. Your phone buzzes as you receive the survey email. When it does, you realize that you weren't thinking about cooking anymore. Instead, you were thinking about a news bulletin that was on the radio moments before about some artisan crafts people coming with the local farmers' market. You were having a good time thinking about how you could go with a friend and buy some new decorations for your home and maybe some gifts for your family. Now imagine yourself being in the situation described here, and respond to all questions accordingly. |
| Summary | How to respond to survey: Please recall your thoughts just before you received the email -- not your thoughts in general during the past hour (or longer). Please be as honest as possible in your response -- there are no right or wrong answers. Please be as precise as possible when using each scale -- carefully select the response that most accurately describes how you felt on each scale of the response. |

*Note.* In the current study, we focused only on the items that were relevant to our primary research question: task-unrelated thought ratings (question 5a), news consumption (question 12a), and motivation ratings (question 4). In a previous paper (Kam et al., 2021), we focused on affective valence. The remaining items on the survey were used for piloting purposes and were not included in the analyses or discussed in the current paper. For each instructions survey item, participants were required to select a response stating, “I understand what the question is asking.” before proceeding. At the end of a survey, they were asked again if they understood the instructions and if not, to email the experimenters for clarification.

^a^ The example used for “extremely off task” was not phrased accurately, as being off-task by engaging in a second task may be considered multitasking. This would have been better phrased as: “…extremely off task if you were thinking about something you saw on the web while you were studying for an exam”. Importantly, all the other “off-task” options accurately portrayed our conceptualization of mind wandering in this study.

^b^ For Study 1, question 12a asked about COVID 19 related news. For Study 2, this question asked about news in general.

^c^ These questions were only presented in Study 2

^d^ The exercise instructions were followed by the same set of survey items again, where participants were required to respond according to what the scenario outlines. Instructions were slightly different for Study 1 and Study 2, where Study 1 specifically focused on COVID-19 related news.

**Supplementary Table 5**

***Descriptive Statistics for Ecological Momentary Assessment Variables***

|  | *n* | TUT  Mean (SD) | Motivation Mean (SD) |
| --- | --- | --- | --- |
| Study 1 |  |  |  |
| Full Sample | 1638 | 2.23 (0.99) | 3.18 (1.02) |
| COVID-19 News | 520 | 2.43 (1.01) | 3.12 (1.03) |
| No COVID-19 News | 1118 | 2.15 (0.97) | 3.21 (1.01) |
| Study 2 |  |  |  |
| Full Sample | 1624 | 2.35 (1.04) | 2.98 (1.07) |
| General News | 118 | 2.53 (0.98) | 2.91 (0.99) |
| No General News | 1436 | 2.33 (1.04) | 2.99 (1.08) |

*Note*. TUT = task unrelated thought, measured on a 4-point Likert scale (1 = completely on-task to 4 = completely off-task). Motivation = motivation for current task, measured on a 5-point Likert scale (1 = not motivated at all to 5 = extremely motivated). COVID-19 News = consumed COVID-19 related news within two hours of EMA survey. No COVID-19 News = did not consume COVID-19 related news within two hours of EMA survey. News = consumed general news media within two hours of EMA survey. No COVID-19 News = did not consume general news media within two hours of EMA survey.

**Supplementary Table 6**

***Model Fit Statistics for Hierarchical Linear Regression Models***

| Predictors | AIC | BIC | Log Likelihood | Deviance | *χ^2^*(1) | *p* |
| --- | --- | --- | --- | --- | --- | --- |
| Study 1 | | | | | | |
| COVID-19 News | 4553.45 | 4580.45 | -2271.72 | 4543.45 |  |  |
| COVID-19 News + Motivation | 4433.26 | 4465.66 | -2210.63 | 4421.26 | 122.18 | < .001 |
| COVID-19 News x Motivation | 4428.17 | 4465.97 | -2207.08 | 4414.17 | 7.09 | .008 |
| Study 2 | | | | | | |
| General News | 4552.13 | 4579.09 | -2271.07 | 4542.13 |  |  |
| General News + Motivation | 4446.44 | 4478.79 | -2217.22 | 4434.44 | 107.69 | < .001 |
| General News x Motivation | 4444.65 | 4482.40 | -2215.33 | 4430.65 | 3.78 | .052 |

*Note.* Each set of rows presents the model fit statistics and comparisons for models of TUT (task- unrelated thought, 1 = completely on-task to 4 = completely off-task) with the inclusion of the corresponding predictor for two separate hierarchical linear regression analyses. Predictors included COVID-19 news (COVID-19 related news consumption, no COVID-19 news consumption as the reference category) for Study 1 or general news (general news media consumption, no general news consumption as the reference category) for Study 2, and task motivation (1 = not motivated at all to 5 = extremely motivated). All models included a random effect of participant on the intercept and a fixed effect covariate for day-in-study (days 1 to 10). Residuals from all models passed assumptions of normality and homogeneity of variance. *Χ^2^* comparisons were calculated for the model indicated in the same row compared to the model in the preceding row. Lower AIC/BIC values indicate better model fit.

AIC = Akaike information criterion. BIC = Bayesian information criterion. Log Likelihood = log likelihood measure of model fit. Deviance = difference in log-likelihoods between the fitted model and a saturated model. *Χ^2^* (1) = chi squared test statistic associated with comparison between nested models (1 degree of freedom). *P* = p-value associated with the chi squared test statistic.

**Supplementary Table 7**

***Simple Effect Analyses of the Interaction between COVID-19 News Consumption and Motivation on TUT (Study 1)***

| Motivation Rating | Estimate | *SE* | 95% CI  [LB, UB] | *t* | *df* | *p* |
| --- | --- | --- | --- | --- | --- | --- |
| 1 - Not Motivated | -0.48 | 0.12 | [-0.72, -0.25] | -4.11 | 1628 | < .001 |
| 2 - Somewhat Motivated | -0.35 | 0.08 | [-0.50, -0.20] | -4.54 | 1586 | < .001 |
| 3 - Moderately Motivated | -0.22 | 0.06 | [-0.33, -0.11] | -4.00 | 1291 | < .001 |
| 4 - Very Motivated | -0.09 | 0.07 | [-0.23, 0.05] | -1.27 | 1345 | .205 |
| 5 - Extremely Motivated | 0.04 | 0.11 | [-0.17, 0.25] | 0.39 | 1519 | .699 |

*Note*. Each row presents the difference in ratings of TUT (task unrelated thought, 1 = completely on-task to 4 = completely off-task), across levels of task motivation (1 = not motivated at all to 5 = extremely motivated), when participants had and had not consumed COVID-19 related news (with no COVID-19 related news consumption as the reference category). The simple effects results reported here were implemented as a follow up to an omnibus linear mixed effects regression model reported in Table 1 for Study 1. Model residuals passed assumptions of normality and homogeneity of variance. The Kenward-Roger method was used to estimate degrees-of-freedom. The Tukey method was used to correct for multiple comparisons.

Estimate = contrast estimate of difference between comparison categories. *SE* = standard error of the contrast estimate. *df* = degrees of freedom for the t-test. 95% CI = 95% confidence interval associated with the contrast estimate. UB = upper bound. LB = lower bound.

**Supplementary Table 8**

***Additional Descriptive Statistics and Spearman Correlations***

|  | Variable | TUT | Motivation | Interest | *M* | *SD* |
| --- | --- | --- | --- | --- | --- | --- |
| Study 1 | | | | | | |
|  | TUT |  |  |  | 2.23 | 0.99 |
|  | Motivation | -.26** |  |  | 3.18 | 1.02 |
|  | Interest | -.36** | .49** |  | 2.82 | 1.14 |
|  | Ease | -.04 | .01 | -.08** | 2.14 | 1.20 |
| Study 2 | | | | | | |
|  | TUT |  |  |  | 2.35 | 1.04 |
|  | Motivation | -.26** |  |  | 2.98 | 1.07 |
|  | Interest | -.34** | .44** |  | 2.63 | 1.16 |
|  | Ease | -.09** | -.11** | -.09** | 2.26 | 1.25 |

*Note*. Correlations are reported for ecological momentary assessment (EMA) ratings as Spearman Rho values. As per a reviewer’s request, these descriptive statistics were reported for questions implemented for piloting purposes for a separate study (except for the motivation question that was reported in the current study). Thoughts just before EMA surveys were received were rated in terms of TUT (task-unrelated thought, 1 = completely on-task to 4 = completely off-task). Current task just before EMA surveys were received were rated in terms of motivation for the task (1 = not motivated at all to 5 = extremely motivated), interest in the task (1 = not interesting at all to 5 = extremely interesting), and task ease (1 = extremely easy to 5 = extremely difficult). All variables except for Motivation and Interest did not pass tests of normality in Study 1. All variables except for Motivation did not pass tests of normality in Study 2. TUT = task-unrelated thought. *M* = mean. *SD* = standard deviation. * *p* < .050. ** *p* < .010.
